# COVID-19 Lockdown Effect on Not Institutionalized Patients with Dementia and Caregivers

**DOI:** 10.3390/healthcare9070893

**Published:** 2021-07-15

**Authors:** Rita Moretti, Paola Caruso, Mauro Giuffré, Claudio Tiribelli

**Affiliations:** 1 Department of Medical Surgical and Health Sciences, Cattinara Hospital Trieste, University of Trieste, Strada di Fiume 447, 34149 Trieste, Italy; paolacaruso83@gmail.com (P.C.); gff.mauro@gmail.com (M.G.); 2 Italian Liver Foundation, Area Science Park, 34100 Trieste, Italy; ctliver@fegato.it

**Keywords:** COVID-19, sVAD, small vessel disease, behavior, caregivers’ burnout, lockdown

## Abstract

SARS-COV-2 is a severe medical condition. Old patients are very vulnerable, but they have been studied only as institutionalized patients. During the lock-down, little attention is dedicated to old, demented patients who lived at home. This study wants to examine their behavioral reactions by video-phone follow-up. We conducted a longitudinal study in subcortical vascular dementia (sVAD) patients. We enrolled 221 sVAD, not institutionalized patients. We divided sVAD patients into low-medium grade sVAD (A) and severe sVAD (B), based on neuroimaging severity degree and executive alterations. At baseline, at the end of lock-down, and two months later, global behavioral symptoms were recorded for each patient. We found significantly higher scores of general behavioral deterioration, anxiety, delusions, hallucinations and apathy after controlling for sVAD severity. The direct consequence was a drastic increment of psychotropic drugs prescribed and employed during the lock-down. Moreover, caregivers’ stress has been evaluated, together with their anxiety and depression levels. During the lock-down, their scores increased and reflected a severe worsening of their behavior. Our data demonstrate that social isolation induces a severe perception of loneliness and abandonment; these fears can exacerbate behavior disturbances in old-aged frail persons. Thus, these can be considered as indirect victims of SARS-COV-2.

## 1. Introduction

The aggressive spread of SARS-COV-2 (COVID-19) has pandemic effects on the world population [1,2,3]. As stated by Wang et al. [4], older and frail people are “the most vulnerable at the onset of natural disaster and crisis.” That is most true for demented people, who, by definition, are the weakest patients. All over the world, and in Italy, it is truest. Institutionalized patients have been victims of pandemic, as brilliantly expressed by Wang et al. [4]: “dementia has emerged as a pandemic in an aging society. The double hit of dementia and COVID-19 pandemics has raised great concerns for people living with dementia”.

Nursing homes and clinical residences have been focused on [5,6] due to the highest chance of infection among older residents. Nevertheless, due to the many urgent contingencies, less attention has been devoted to older demented people who live in their homes and have been isolated. As well-written, “social distancing measures have been adopted worldwide to contain the spread of transmission of the COVID-19…the older adults, and especially those with cognitive impairments under home care, are particularly vulnerable to the disruption caused by social distancing” [7]. As far as old age is a complicated status of life, many factors can modify its development. The possible contribution of multiple biological events cannot be neglected, particularly the underlying influence of chronic diseases and geriatric conditions, per se, which might compromise the cognitive functions and the pathological conditions [8]. Therefore, we decided to study the behavior of home-isolated vascular demented patients and their caregivers, during the lockdown, by weekly programmed video-phone interviews, taking care of the recommendations by Alzheimer’s Disease International [9].

## 2. Methods

We conducted a longitudinal study in a Neurological group of patients affected by subcortical vascular dementia (sVAD) and followed up by our Unit, Complex Neurological Cases of the University of Trieste. From 10 March 2020 to 18 May 2020, we enrolled 268 sVAD, previously diagnosed patients, not institutionalized. Forty-seven patients have been excluded due to the absence of an informed caregiver. The diagnosis of subcortical vascular dementia has been previously made, following the NINDS AIREN criteria [10,11,12]. sVaD was diagnosed when the CT/MRI scan showed moderate to severe ischemic white matter changes and at least one lacunar infarct [13,14]. All the patients had severe white matter hyperintensities on MRI, localized around the lateral ventricles or within the deep white matter [15,16]. Brain CT-scans (78% of cases, when prescribed by General Practitioners) and MRI images (100% of cases) were available for all the patients; a neurologist (RM) revised all the imaging, employing the Blennow scale for CT scans [17,18] and the Fazekas scale for MRI imaging [19] following parameters of recent literature [20,21]. We applied a standardized algorithm to define the extension of the altered white matter and the number of lacunar events, according to the Fazekas’ Scale as a 4-level classification: we excluded all the periventricular white matter alterations (PWM), which traditionally seems related to a combination of demyelination, ependymitis granularis and subependymal gliosis, as well as small vessel ischemia [22]. On the contrary, we employed the Fazekas scale for deep white matter alterations DWM [22]. Therefore, we employed the following parameters: 0 = absent DWM alterations; 1 = punctate foci of DWM alterations; 2 = beginning confluence of DWM alterations; 3 = large confluent areas of DWM alterations.

There was a 93.8% inter-rater agreement (RM and PC) for the independent assessment of the scans (kappa = 0.79). Patients were not included in the study if they showed signs of normal pressure hydrocephalus, previous brain tumors and the previous diagnosis of major cerebrovascular event, white matter lesions, caused by different specific etiologies, such as multiple sclerosis, collagen vascular disease and genetic forms of vascular dementia (such as CADASIL or CARASIL). Patients with previous major psychiatric illnesses (i.e., schizophrenia, bipolar disorders, psychosis, compulsive-obsessive disorders) or central nervous system disorders and alcoholism were excluded. Their average neurological follow-up before the lockdown was of 17.4 (3.2) months.

We finally evaluated 221 sVAD diagnosed patients, not institutionalized. Every week, the patients and their caregivers received a video call, utilizing commonly accessible mobile communication platforms (WhatsApp and Facetime) by RM; the caregiver should assist the video call. Such video-call facilitate the engagement with the patients and with their relatives. In the video call, the patients underwent a general behavioral assessment (with specific tests) and a Quality of life (QUALID) assessment. The caregivers were tested at the same time with three tests (see in the following paragraph). With the finding out of eventual severe behavioral alterations, RM decided to modify or implement a specific therapy; each modification has been enclosed in the patients’ files. The caregivers were asked to report body temperature each day, and any suspicious signs, i.e., cough, mucus or phlegm, sore throat, loss of smell or taste, dyspnea, diarrhea. If present and persistent, they should contact their general practitioner or emergency ward. A video call has been scheduled weekly during the period in-between 10 March 2020 and 18 May 2020. After the lockdown period, the patients and their caregivers have been contacted again with a weekly video call, undergoing the same tests, till two months after the end of the lockdown, 18 July 2020. All of them underwent the same tests, registered during the video call. We present the averages scores obtained by our patients and caregivers before 10 March 2020, at the end of the lockdown, 18 May 2020, and two months later, 18 July 2020.

The present study was conducted following the Declaration of Helsinki and the Ethics Guidelines of the Committee of the University-Hospital of Trieste, CEUR, point 4.

### 2.1. Neuropsychological Instruments

All the patients have been previously carefully studied.

The primary outcomes of this study for sVAD patients during the lock-down period and two months after the end of it were:

1. Global behavioral symptoms, assessed by the Neuropsychiatric Inventory, NPI [23].
2. NPI specific scores, depression, hallucinations and delusions have been registered (Frequency and intensity of symptoms, with the correct score of 4 × 3, considering a maximum score of 12) [23].
3. Anxiety, assessed by the Hamilton Anxiety Rating Scale (HAM-A) (score: 0–56; a total score comprised 0–17, estimated mild anxiety; 18–24: mild to moderate anxiety; 25–30: severe anxiety) [24].
4. Apathy and abulia, assessed by the Apathy Evaluation Score (AES-C) (clinical examination; score, 18–72; higher scores reflect a higher level of apathy) [25].
5. The quality of life, assessed by the Quality of Life in late-stage Dementia Scale (QUALID) [26]; the proxy rating scale consists of 11 items that are rated on a five-point scale. The items are rated by frequency of occurrence, comprising both positive and negative dimensions of concrete and observable mood and performance. Scores are summed to range from 11 to 55. A lower score indicates a higher quality of life.

The caregivers have been tested with:

1. The caregiver stress was assessed by the Relative Stress Scale, RSS (score 0–60; higher scores reflect more caregiver stress) [27].
2. Beck’s Depression Inventory (version for Italian population) (score: 0–39; total score 10–19 indicated mild depression; total score 20–29 indicated moderate depression; >30 indicated severe depression) [28,29].
3. Anxiety, assessed by the Hamilton Anxiety Rating Scale (HAM-A) (score: 0–56; a total score comprised 0–17, estimated mild anxiety; 18–24: mild to moderate anxiety; 25–30: severe anxiety) [24].

Drugs have been scheduled for each patient, and each variance has been registered during the follow-up.

General conditions and potential COVID-19 symptoms have been reported.

### 2.2. Statistical Analysis

Statistical analyses were performed using the Statistical Package for the Social Sciences (SPSS, version 17.6). The difference in the baseline, at the end of lock-down (69 days later) and two months later (18 July) in sVAD patients was assessed by one-way repeated measures ANOVA test for the continuous variable, obtained by video-phone interviews. After the general results, we further divided the groups according to pre-lockdown severity; we further examined the results in the three temporal times described above with a one-way repeated measures ANOVA test. If the ANOVA results were found significant, the Tukey test also did the multiple comparison analysis to examine these groups, which were significantly different from each other.

A comparison of mean values obtained by the two groups in the three temporal occasions has been obtained by a two-way repeated measures ANOVA to compare the longitudinal changes of variables between group A and B.

We performed a multivariate linear regression analysis to evaluate the relationship between all the recruited patients and behavioral results. In Model 1, we adjusted for sex, age, race and educational levels; in Model 2, we further adjusted for disease severity level (based on prior lock-down average scores of FAB [28,29] and Fazekas scale [19]. Prescribed drugs have been reported in the two groups during the lock-down period, at the end of it and two months later.

We finally reported the data obtained by caregivers’ test scores; the difference in the baseline, at the end of lock-down (69 days later) characteristics and two months later (18 July) in caregivers was assessed by one-way repeated measures ANOVA test for the continuous variable, obtained by video-phone interviews. Results are presented as mean with standard deviations, and *p* values are presented where appropriate.

## 3. Results

The demographic variables, i.e., age, gender and educational levels, have been reported in Table 1; one-way repeated measures analysis of variance (ANOVA) method was applied to explore the statistical difference among mean value in the three groups with our data, at the first video-visit at the beginning of the lock-down, 10 March, at the end of it, 18 May, and two months later (18 July) (Table 2). The five different behavioral variables (NPI, HAM-A, AES-C, RSS; QUALID and sub-scores of NPI, NPI Hallucinations, NPI delusions and NPI depression) studied were significantly different (*p* < 0.01) in the examined patients (Table 2). Two months after the end of the lock-down, there was a slight but significant decrease in the examined neuropsychological scores (Table 2). No recruited patients did show any sign of COVID-19 infection during the entire follow-up.

We decided to divide our sVAD patients into two groups: a low-medium grade of sVAD (Group A) and the other with a severe score of SVAD (Group B). The division has been obtained by two average pre-lockdown neuropsychological parameters: FAB [27,28] and Fazekas scale [19]. Arbitrarily, low-medium sVAD status is represented by an average FAB scores 8–14, associated with an average Fazekas scores 1–2; on the contrary, severe sVAD is represented by average FAB scores 4–7, average Fazekas scores of 2–3 (see Table 3). Group A was composed of 146 and Group B of 75 patients.

We then divided the obtained global results, dividing the entire population into two groups: Table 4 reported the repeated measures analysis of variance (ANOVA) in Group A in the three periods. Table 5 reported the results obtained by group B.

Table 6 reported the two-way repeated measures ANOVA to compare the longitudinal changes of variables between group A and B. Pre-lockdown, the differences between the groups are dramatically significant, and they maintained this way after the lockdown and two months later the end of it (apart from depression NPI sub-scores). Results changed significantly during the lockdown, and the scores declined at the end of it and two months later.

Moreover, as reported in Table 4, Table 5 and Table 6, delusions and hallucinations were rarely reported before the lockdown in the two groups (differences are not significant, as reported in Table 6); they became more common during lockdown in both groups, even if worse in Group B (Table 5 and Table 6) and they decreased significantly after two months the lockdown ending (Table 6).

We performed a multivariate linear regression analysis to evaluate the relationship between lockdown limits and social constraints, subcortical vascular dementia and behavioral impairment during the lockdown period. In Model 1, considering all the patients together, we adjusted for sex, age and educational level, and in Model 2, we further adjusted for a grade of severity of sVAD (NPI and Fazekas) (Table 7). NPI, HAM-A, AES-C and RSS failed to have the worst scores concerning age, sex and educational level (Model 1); on the contrary, we have found significantly lower scores of NPI, HAM-A, AES-C and RSS after controlling for sVAD severity, as seen in Model 2 (Table 7).

According to our observation, all the patients took more benzodiazepines and more typical and atypical neuroleptics during the lockdown period. In particular, lower-medium grade sVAD patients were prescribed benzodiazepines (98%), typical (31%) and atypical neuroleptics (38%); 19% of them have been prescribed two drugs at the same time. Severe sVAD patients were prescribed benzodiazepines (92%), 43% typical, 71% atypical neuroleptics and 49% of them have prescribed two drugs simultaneously (Table 6). Two months later, there is a reduction of prescribed drugs in both groups. In patients with low-medium sVAD, 41% have been prescribed benzodiazepine, 16% typical and 21% atypical neuroleptics and 10% two or more drugs. In the severe form of sVAD, 43% of patients have been prescribed benzodiazepines, 30% typical and 51% atypical neuroleptics, and 15% to drugs simultaneously (Table 8). These results are pretty encouraging compared to the end of the lockdown but very impressive compared to the previous lockdown prescriptions.

**Figure 1:**
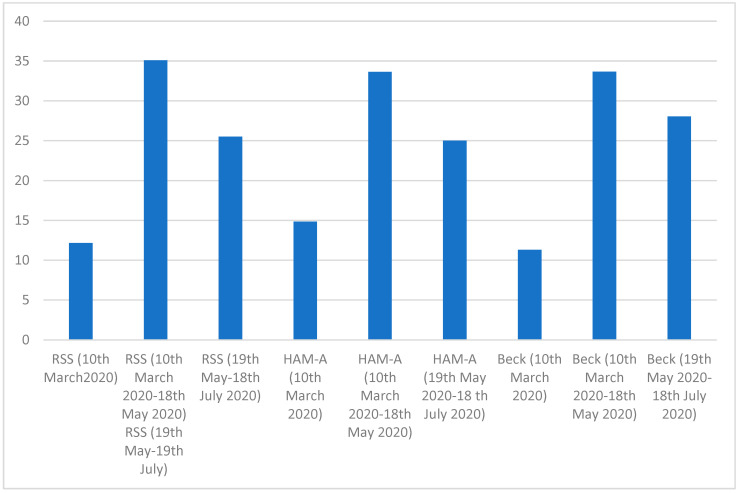
Graphical Assessment of the Caregivers behavioral alterations during the follow-up.

One-way repeated-measures analysis of variance (ANOVA) method was applied to explore the statistical difference among mean value in the caregivers, at the first video-visit at the beginning of the lock-down, 10 March, at the end of it, 18 May, and two months later (18 July) (Table 9). The three different behavioral variables (RSS, HAM-A and Beck’s depression test) studied were significantly different (*p* < 0.01) in the examined healthy subjects (Table 9). Two months after the end of the lockdown, there was a significant decrease in the examined neuropsychological scores (Table 9 and Figure 1).

## 4. Discussion

Our study examined the indirect effect of coronavirus disease 19 (COVID-19) lockdown on behavioral and psychological symptoms of dementia.

This longitudinal study has several limitations: it covers a limited number of home resident-sVAD people, and it stands on video-calls interviews, in the beginning, at the end of the lockdown and two months later. However, to the best of our knowledge, it is the first study that directly approaches at the same time patients (only including sVAD) and their caregivers to obtain information on their behavioral condition under strained lockdown. We demonstrated a significant increase in behavioral disturbances (as expressed by the significant increment of NPI, specifically by three subscores of it: delusions, hallucinations and depression). Aggressiveness and anxiety increased significantly (testified by NPI and by Hamilton Anxiety scores). There is a slight but significant increase in apathy, as tested by AES-C [30]. The worst effects can be observed in patients with the severest form of sVAD before the lockdown.

On the other hand, caregivers’ stress is the most direct and manifest result of the tumultuous increase of the patients’ behavioral disorders; so, they developed signs of exhaustion and burnout after the 69 days of lockdown, which slightly decreased in the following two months.

We have suggested, during the entire period of lockdown, some non-pharmacological strategies (i.e., using memory aids, simplifying daily routines, evaluating and adjusting amplification hearing aids, and evaluating and adjusting sleep routines, using a night light, etc.) [31,32,33] however, they were not influential. The essential behavioral alterations required the need for drugs, testified by an increment of prescribed benzodiazepines, typical or atypical neuroleptics, during the lockdown. They slightly decreased after the end of it.

Our conclusions demonstrated that social distancing likely had exacerbated the impact of social isolation, determined by mobility limitation, the feeling of loneliness and a wide reduction of face-to-face social interactions. Social isolation might cause the feeling of loneliness, which can exacerbate behavior alterations. These conclusions align with those found in a very recent work, which assessed the NPI and the revised University of California at Los Angeles (UCLA) loneliness scale [34]. The revised UCLA loneliness scale score was not significantly associated with age, education, Mini-Mental state examination score, gender, physical impairments and marital status. On the contrary, this score was a significant predictor of NPI delusion and hallucination subscale scores and depression scores. The Authors concluded that loneliness is an effective risk for behavioral alterations, especially depression and psychotic symptoms. Another recent and important [35] concluded that loneliness is associated with an increased risk of developing dementia, and it could be considered one of the modifiable factors that can be intervened to reduce dementia risk. It is known that poor social engagement indices were associated with increased dementia risk, including having a poor social network and poor social support, whereas good social engagement was modestly protective [36]. Social isolation is per se a devastating condition, even for healthy subjects, but even worse for demented people [37,38]

Therefore, this even-limited experience should make us reflect on the indirect damages produced by COVID-19 pandemic; any of the included patients and their relatives have been infected, but signs of their life- experience, in terms of behavioral disturbances and burnout, are tangible. The anticipated recognition of neuropsychiatric symptoms and visual hallucinations may be necessary concerning optimizing care and determining prognosis, or at least, to reduce their consequence in an actual frail population [39]. The successful management of troublesome behaviors associated with vascular dementia can significantly improve the overall quality of life. Finding effective social and welfare networks, correct instructions and tangible help for their caregivers are likely to impact patient care, caregiver distress and institutionalization substantially [40]. Unfortunately, during this lockdown, the frailest patients, due to their constant need for community services and their interpersonal links, suffered more and more and cannot make relevant life adjustments to the suddenly changed environment [41]. Therefore, they could be counted as indirect victims of COVID-19.

## Figures and Tables

**Table 1:** Baseline characteristics of the population before the lock-down period.

Characteristics	Mean (SD)
Age (years)	75.6 (6.6)
Male/female	102/119
Educational level (yrs school)	12.1 (2.6)
Time of follow-up (months) before 10 March 2020	17.4 (3.1)

**Table 2:** Comparison of mean value and SD of the defined neuropsychological scores.

Variables	10 March	18 May	18 July	F Chi2 Value	DF	*p* Value
NPI (0–144)	16.3 ± 2.1	36.4 ± 5.3	30.1 ± 2.3	0.91	2.77	0.01
HAM-A (0–56)	19.1 ± 2.7	39.7 ± 7.1	34.6 ± 5.2	0.89	2.65	0.01
AES-C (18–71)	21.3 ± 3.4	31.4 ± 2.5	28.3 ± 2.7	0.78	2.31	0.01
QUALID (11–55)	24.5 ± 2.7	35.4 ± 3.7	26.1 ± 3.1	0.97	2.4	0.01
NPI Hallucinations (0–12)	3.5 ± 0.3	11.2 ± 0.6	7.7 ± 4.1	0.76	2.36	0.01
NPI Delusions (0–12)	0.9 ± 0.2	11.3 ± 0.7	8.3 ± 0.6	0.67	2.17	0.01
NPI Depression (0–12)	9.6 ± 1.1	10.9 ± 0.8	9.7 ± 0.9	0.78	2.34	0.05

**Table 3:** Parameters (mean value and SD) of the defined neuropsychological scores in our patients, before 10 March 2020.

Variables	Low-Medium sVAD (146 pts) Group A	Severe sVAD (75 pts) Group B	F Chi2 Value	DF	*p* Value
FAB	12.3 ± 3.4	9.5 ± 2.7	0.91	2.07	0.01
Fazekas Scale	1.9 ± 1.6	2.7 ± 0.1	0.98	2.65	0.01

**Table 4:** Comparison of mean value and SD of the defined neuropsychological scores in Group A.

Variables	10 March	18 May	18 July	F Chi2 Value	DF	*p* Value
NPI (0–144)	11.3 ± 1.1	31.1 ± 0.3	28.4 ± 1.1	0.76	2.4	0.01
HAM-A (0–56)	17.3 ± 1.2	32.3 ± 3.1	27.1 ± 3.1	0.91	2.3	0.01
AES-C (18–71)	18.3 ± 1.2	27.1 ± 1.3	25.1 ± 1.8	0.87	2.4	0.01
QUALID (11–55)	21.2 ± 1.4	30.1 ± 1.2	24.2 ± 1.3	0.69	2.1	0.01
NPI Hallucinations (0–12)	3.1 ± 0.1	8.1 ± 1.1	6.9 ± 1.2	0.56	2.1	0.01
NPI Delusions (0–12)	0.7 ± 0.1	7.8 ± 1.2	5.3 ± 0.3	0.67	2.2	0.01
NPI Depression (0–12)	8.1 ± 0.6	10.1 ± 0.3	8.7 ± 0.4	0.89	2.1	0.05

**Table 5:** Comparison of mean value and SD of the defined neuropsychological scores in Group B.

Variables	10 March	18 May	18 July	F Chi2 Value	DF	*p* Value
NPI (0–144)	24.1 ± 1.6	39.1 ± 3.1	32.4 ± 1.5	0.93	2.5	0.01
HAM-A (0–56)	21.3 ± 1.1	43.1 ± 2.5	37.2 ± 2.3	0.88	2.4	0.01
AES-C (18–71)	25.1 ± 1.7	36.1 ± 1.2	32.1 ± 1.5	0.98	2.4	0.01
QUALID (11–55)	26.1 ± 1.2	37.3 ± 2.1	29.7 ± 1.2	0.79	2.3	0.01
NPI Hallucinations (0–12)	4.2 ± 0.6	11.5. ± 0.5	9.7 ± 2.1	0.76	2.6	0.01
NPI Delusions (0–12)	1.3 ± 0.3	11.6 ± 0.1	9.1 ± 0.4	0.67	2.5	0.01
NPI Depression (0–12)	9.7 ± 0.7	11.1 ± 0.8	9.4 ± 0.5	081	2.4	0.05

**Table 6:** Comparison of mean value and SD to compare the longitudinal changes of variables between group A and B.

Variables	10 March Group A	10 March Group B	F Chi2 Value	DF	*p* Value
NPI (0–144)	11.3 ± 1.1	24.1 ± 1.6	0.87	2.5	0.01
HAM-A (0–56)	17.3 ± 1.2	21.3 ± 1.1	0.89	2.4	0.01
AES-C (18–71)	18.3 ± 1.2	25.1 ± 1.7	0.91	2.4	0.01
QUALID (11–55)	21.2 ± 1.4	26.1 ± 1.2	0.83	2.3	0.01
NPI Hallucinations (0–12)	3.1 ± 0.1	4.2 ± 0.6	0.87	2.6	0.057
NPI Delusions (0–12)	0.7 ± 0.1	1.3 ± 0.3	0.91	2.5	0.056
NPI Depression (0–12)	8.1 ± 0.6	9.7 ± 0.7	0.87	2.4	0.05
**Variables**	**18 May** **Group A**	**18 May** **Group B**	**F Chi2 Value**	**DF**	***p*** **Value**
NPI (0–144)	31.1 ± 0.3	39.1 ± 3.1	0.78	2.5	0.05
HAM-A (0–56)	32.3 ± 3.1	43.1 ± 2.5	0.93	2.4	0.01
AES-C (18–71)	27.1 ± 1.3	36.1 ± 1.2	0.94	2.4	0.01
QUALID (11–55)	30.1 ± 1.2	37.3 ± 2.1	0.81	2.3	0.05
NPI Hallucinations (0–12)	8.1 ± 1.1	11.5 ± 0.5	0.83	2.6	0.01
NPI Delusions (0–12)	7.8 ± 1.2	11.6 ± 0.1	0.84	2.5	0.01
NPI Depression (0–12)	10.1 ± 0.3	11.1 ± 0.8	0.91	2.4	0.056
**Variables**	**18 July** **Group A**	**18 July** **Group B**	**F Chi2 Value**	**DF**	***p*** **Value**
NPI (0–144)	28.4 ± 1.1	32.4 ± 1.5	0.91	2.5	0.01
HAM-A (0–56)	27.1 ± 3.1	37.2 ± 2.3	0.79	2.4	0.01
AES-C (18–71)	25.1 ± 1.8	32.1 ± 1.5	0.93	2.4	0.01
QUALID (11–55)	24.2 ± 1.3	29.7 ± 1.2	0.87	2.3	0.01
NPI Hallucinations (0–12)	6.9 ± 1.2	9.7 ± 2.1	0.89	2.6	0.01
NPI Delusions (0–12)	5.3 ± 0.3	9.1 ± 0.4	0.82	2.5	0.01
NPI Depression (0–12)	8.7 ± 0.4	9.4 ± 0.5	0.85	2.4	0.05
**Variables**	**10 March (*p* Value)**	**18 May (*p* Value)**		**18 July (*p* Value)**	
NPI (0–144)	−12.8 ± 0.5 (*p* < 0.01)	−8 ± 2.8 (*p* < 0.01)		−4 ± 0.4 (*p* < 0.05)	
HAM-A (0–56)	−4 ± 0.1 (*p* < 0.05)	−10.8 ± 0.6 (*p* < 0.01)		−10.1 ± 0.8 (*p* < 0.01)	
AES-C (18–71)	−6.8 ± 0.5 (*p* < 0.01)	−9 ± 0.1 (*p* < 0.01)		−7 ± 0.3 (*p* < 0.01)	
QUALID (11–55)	−4.9 ± 0.2 (*p* < 0.05)	−7.2 ± 0.9 (*p* < 0.01)		−5.5 ± 0.1 (*p* < 0.01)	
NPI Hallucinations (0–12)	−1.1 ± 0.5 (NS)	−3.4 ± 0.6 (*p* < 0.01)		−2.8 ± 0.9 (*p* < 0.01)	
NPI Delusions (0–12)	−0.6 ± 0.2 (NS)	−3.8 ± 0.9 (*p* < 0.01)		−3.8 ± 0.1 (*p* < 0.01)	
NPI Depression (0–12)	−0.3 ± 0.1 (NS)	1 ± 0.5 (*p* < 0.5)		0.7 ± 0.1 (NS)	

**Table 7:** Association between sVAD and neuropsychological variable with a multivariate linear regression analysis.

	B	*p* Value	SE	95% CI
NPI				
Model 1	0.12	0.56	3.56	0.2–0.9
Model 2	0.88	0.01	3.12	0.31–1.18
HAM-A				
Model 1	0.37	0.47	3.43	0.2–0.7
Model 2	0.72	0.01	3.17	0.21–2.19
AES-C				
Model 1	0.24	0.43	2.67	0.3–2.4
Model 2	0.94	0.01	3.21	0.5–2.8
RSS				
Model 1	0.43	0.54	2.78	0.1–4.9
Model 2	0.88	0.01	2.12	0.9–2.9
QUALID				
Model 1	0.56	0.78	2.34	0.4–3.4
Model 2	0.71	0.01	2.89	0.6–2.8

**Table 8:** Drugs variants during lock-down (reported patients prescribed and their %).

Drugs	10 March 2020		18 May 2020		18 June 2020	
	Group A	Group B	Group A	Group B	Group A	Group B
Benzodiazepines	44 (30%)	13 (17%)	59 (41%)	32 (43%)	59 (41%)	32 (43%)
Typ. Neurolep.	19 (13%)	19 (25%)	27 (16%)	22 (30%)	27 (16%)	22 (30%)
Atyp. Neurolep.	16 (11%)	36 (48%)	30 (21%)	38 (51%)	30 (21%)	38 (51%)
Two drugs together	0	7 (9%)	28 (19%)	37 (49%)	14 (10%)	11 (15%)

**Table 9:** Comparison of mean value and SD of the caregivers’ parameters.

Variables	10 March	18 May	18 July	F Chi2 Value	DF	*p* Value
RSS (0–66)	12.15 ± 1.1	35.1 ± 1.3	25.5 ± 1.9	0.98	2.77	0.01
HAM-A (0–56)	14.9 ± 2.3	33.7 ± 2.1	25.1 ± 3.2	0.82	2.61	0.01
Beck	11.3 ± 1.2	33.7 ± 1.4	28.1 ± 1.3	0.67	2.56	0.01

## Data Availability

Not applicable.
